# Publication, publication, publication…

**DOI:** 10.1308/003588414X13814021676918

**Published:** 2014-01

**Authors:** Colin Johnson

2013 has been a year of relative stability at the *Annals*. After two years of rapid changes in staff and implementation of our electronic manuscript handling, this year we had time and people to consolidate. Authors in particular will have noticed the hard work of Kim Lewry, our Editorial Assistant, who has tracked down papers that were stuck in the system and has made a substantial contribution to the reduced times for processing submissions. We have also benefitted from the steady hand of Matthew Whitaker, Publications Manager, who has ensured that our systems meet the needs of editors and authors. During this year, Matthew has overhauled the OJS manuscript handling system and we hope that contributors are seeing the benefits. Our team of section editors and the Editorial Board have all helped ensure the speedy and thorough assessment of submitted papers.

A major issue for the editors this year has been publication ethics. We take very seriously our obligation to maintain the highest possible standards. The *Annals* is affiliated to the Committee on Publication Ethics (COPE), which publishes guidance for journals, editors and authors. Prospective authors should read and adhere to the principles expressed in the COPE document on international standards for authors (http://publicationethics.org/international-standards-editors-and-authors).

Readers will recall that earlier in 2013 we detected (before publication) a case of attempted dual publication. The relevant deanery was informed of the actions of the UK trainees concerned. In this issue we have announced retraction of a paper that seems to be unreliable. We have informed the relevant registration authorities.

Publication ethics is discussed in the new College course *How to Write a Surgical Paper*, which we have established over the last year, working with Professor Derek Alderson, Council Member and Editor-in-Chief of the *British Journal of Surgery (BJS)*. Based on the successful *BJS* course, this one-day course gives guidance and practical training in scientific writing, and has proved popular. The next course runs in March 2014.

The *Annals* continues to receive increasing numbers of submissions. In 2010 we received 196 original research papers and accepted 57 (29%). In the 12 months to October 2013 we received 367 submissions and have accepted 60. Some of the papers still in review will also be accepted but the final acceptance rate for 2013 will be lower than previously. This has been achieved with a substantial reduction in the mean time taken to review, from 68 days in 2010 to 38 days in 2013. I am grateful to all our reviewers (and to Kim Lewry) for this tremendous improvement in the service we offer to authors. We hope that this will help to attract more high quality papers.

In order to promote access to our published papers, and to meet the requirements of UK and other funders, from January 2014 the *Annals* will be a hybrid open access journal. This means that authors will have the option to have their article made freely available to all immediately on publication, on both our own platform and on PubMed, by payment of an article processing charge (‘gold’ open access). All other *Annals* papers will be published under a ‘green’ open access model, meaning that the papers will be made freely available to all after an embargo period of one year. Our policies are compliant with current Research Councils UK and Wellcome Trust guidelines on open access, and are part of the College’s ongoing commitment to extending the frontiers of surgery through clinical research. A summary of our open access policy appears on page v and you can read more, including details about licensing and pricing, on our website: http://www.rcseng.ac.uk/publications/open-access.

Looking forward, I am not making specific predictions for 2014. However, I am confident that digital publishing will reach us all in the near future. The publishing paradigm has changed because of improved technology and the wide availability of handheld reading devices. Paper journals have always been seen as convenient, transportable, delivered to the doormat or desk and available at any time. A digital resource on a publisher’s website (requiring a password, connection to the internet and, crucially, the time and effort to go and look at it) always seemed too cumbersome for day-to-day use. It is the difference between reading the current issue between cases in theatre and going to the library to look up a specific topic.

I believe we are now at a tipping point, with most of us able to receive electronic content on a Kindle, iPad® or other device. Content is delivered without effort on our part and is then carried easily, to be accessible when we want to read it. I can look at the Sunday paper before I get out of bed, then read some more over coffee in the beach café. I can go back to finish an interesting article later in the week. I now have no use for a printed newspaper (except for lighting the fire!). Surgical journals are also moving towards this digital delivery format and because this is more convenient, it will rapidly make paper redundant for many of our readers.

How quickly we move to digital delivery, and what other changes may be made to the content and format of the *Annals*, will depend in some measure on the responses we receive to surveys of authors and readers, the latter of which is live now (http://bit.ly/rcsreadersurvey). Your views are important – please respond as fully as you can. Our main aim will always be to provide an interesting clinical journal, relevant to the work of Fellows, Members and other surgeons, in every surgical discipline and any country. We welcome your contributions and hope for your continuing support.

